# A phosphatidylinositol phosphate kinase inhibits Ras activation and regulates chemorepulsion in *Dictyostelium discoideum*

**DOI:** 10.1242/jcs.260541

**Published:** 2023-07-27

**Authors:** Sara A. Kirolos, Chance E. Hatfield, Ryan J. Rahman, Kristen M. Consalvo, Nolan K. Dittenhauser, Richard H. Gomer

**Affiliations:** Department of Biology, Texas A&M University, 301 Old Main Drive, College Station, TX 77843-3474, USA

**Keywords:** AprA, *Dictyostelium discoideum*, Phagocytosis, Phosphatidylinositol, Ras, Chemorepulsion

## Abstract

During developmental and immune responses, cells move towards or away from some signals. Although much is known about chemoattraction, chemorepulsion (the movement of cells away from a stimulus) remains poorly understood. Proliferating *Dictyostelium discoideum* cells secrete a chemorepellent protein called AprA. Examining existing knockout strains, we previously identified proteins required for AprA-induced chemorepulsion, and a genetic screen suggested that the enzyme phosphatidylinositol phosphate kinase A (PIPkinA, also known as Pik6) might also be needed for chemorepulsion. Here, we show that cells lacking PIPkinA are not repelled by AprA, and that this phenotype is rescued by expression of PIPkinA. To bias cell movement, AprA inhibits Ras activation at the side of the cell closest to the source of AprA, and we find that PIPkinA is required for AprA to inhibit Ras activation. PIPkinA decreases levels of phosphatidylinositol 4-phosphate [PI(4)P] and phosphatidylinositol (3,4,5)-trisphosphate [PI(3,4,5)P_3_], and possibly because of these effects, potentiates phagocytosis and inhibits cell proliferation. Cells lacking PIPkinA show normal AprA binding, suggesting that PIPkinA regulates chemorepulsion at a step between the AprA receptor and AprA inhibition of Ras activation.

## Teaser

**Highlighted Article:** This study expands upon the role of *Dictyostelium* PIPkinA, furthering our understanding of the underlying mechanism of chemorepulsion and informing development of therapeutics for acute respiratory distress syndrome.

## INTRODUCTION

Biased cell migration is essential for the induction of development, morphogenesis and immune responses (Macabenta and Stathopoulos, 2019; Scarpa and Mayor, 2016; Hampton and Chtanova, 2019). Much of our understanding of eukaryotic chemoattraction, the movement of cells towards the source of a diffusible attractant chemical, has come from studies using the unicellular eukaryote *Dictyostelium discoideum*. During development, *D. discoideum* cells aggregate using chemoattraction to relayed pulses of cyclic adenosine monophosphate (cAMP) (Garcia and Parent, 2008; Saran et al., 2002; Singer et al., 2019; De Palo et al., 2017). Five major pathways downstream of the cAMP receptor cAR1 (encoded by *carA*), including the mammalian target of rapamycin complex 2 (mTORC2), guanylyl cyclase, mitogen-activated protein kinase (MAPK), phosphoinositide 3-kinase (PI3K)–phosphatidylinositol (3,4,5)-trisphosphate [PI(3,4,5)P_3_] and phospholipase A (PlA) pathways, appear to act synergistically to drive chemoattraction towards cAMP (Chen et al., 2007; Kim et al., 1998; Veltman et al., 2016; Nichols et al., 2019; Postma et al., 2003; Stepanovic et al., 2005; Van Haastert, 2010).

Chemorepulsion is the biased movement of cells away from a signal. We have previously found an endogenous chemorepellent secreted by proliferating *Dictyostelium* cells called autocrine proliferation repressor protein A (AprA) (Phillips and Gomer, 2012). In a colony of growing cells, the extracellular AprA concentration will be high within the colony and low outside the colony, creating a gradient. This AprA gradient causes cells at the edge of a colony to move away from the colony, possibly to find new sources of food (Phillips and Gomer, 2012; Kirolos and Gomer, 2022).

Cells such as *Dictyostelium* and neutrophils move by extending a pseudopod, attaching the pseudopod to the substrate and then essentially squeezing the cell into the pseudopod (Devreotes and Zigmond, 1988; Ananthakrishnan and Ehrlicher, 2007; Uchida and Yumura, 2004; Eidi, 2017; Cooperet al., 2012). In the absence of guidance cues, cells move in random directions (Cramer et al., 2018). A localized activation of Ras initiates pseudopod formation (van Haastert et al., 2017; Kirolos and Gomer, 2022). AprA inhibits Ras activation and thus pseudopod formation at the region of the cell closest to the source of the AprA, causing the normal random cell motility to be biased away from the AprA source (Kirolos and Gomer, 2022; Rijal et al., 2019). Major proteins involved in the AprA-induced chemorepulsion pathway include the G-protein-coupled AprA receptor GrlH, the Gβ (GbpA) and Gα8 (GpaH) G protein subunits, protein kinase A, components of the mTORC2 complex, phospholipase A (PlaA), Erk1 (also known as ErkA), p21-activated protein kinase D (PakD), and the Ras proteins RasC and RasG (Bakthavatsalam et al., 2009; Phillips and Gomer, 2014; Rijal et al., 2019; Tang et al., 2018; Wu et al., 1995). Unlike chemoattraction towards cAMP, the PI3K–Akt/protein kinase B and guanylyl cyclase pathways are not required for AprA-induced chemorepulsion (Kortholt et al., 2011; Ma et al., 1997; Rijal et al., 2019).

In previous work to identify additional proteins in the AprA-induced chemorepulsion pathway, we utilized restriction enzyme-mediated integration (REMI; Kuspa and Loomis, 1992) to generate random insertional mutants (Kirolos et al., 2022). In a partial screen of these mutants, we found 26 insertions that disrupted AprA-induced chemorepulsion, and for 17 of these, the sequences of the genomic DNA at the insertion sites identified the location of the REMI insertion, and thus possibly the gene that when disrupted by REMI insertion caused a loss of AprA-induced chemorepulsion (Kirolos et al., 2022). One of the REMI insertions was in the gene encoding phosphatidylinositol phosphate kinase A (PIPkinA, also known as Pik6), a protein expressed during early development and throughout aggregation (Guo et al., 2001). Type 1 phosphatidylinositol phosphate kinases convert phosphatidylinositol 4-phosphate [PI(4)P] to phosphatidylinositol (4,5)-bisphosphate [PI(4,5)P_2_], while the type 2 enzymes convert phosphatidylinositol 5-phosphate to PI(4,5)P_2_ (Katan and Cockcroft, 2020). PIPkinA has 32% amino acid sequence similarity to human phosphatidylinositol 4-phosphate 5-kinase 1 (PIP5KIα, also known as PIP5K1A) and 28% similarity to human phosphatidylinositol 4-phosphate 5-kinase 2 (PIP5KIIα, also known as PIP4K2A) (Guo et al., 2001; Yoshida et al., 1994; Boronenkov and Anderson, 1995; Mikami et al., 1998). Both PIP5KIα and PIP5KIIα convert PI(4)P to PI(4,5)P_2_ (Amos et al., 2019; Fedorenko et al., 2008). *Dictyostelium* PIPkinA contains an enzymatically active conserved phosphatidylinositol phosphate kinase domain (Guo et al., 2001) that catalyzes the conversion of PI(4)P to PI(4,5)P_2_ (Guo et al., 2001). PI(4,5)P_2_ can act directly as a second messenger or act as a precursor to generate other second messengers, such as inositol (1,4,5)-trisphosphate (IP_3_), diacylglycerol (DAG) or PI(3,4,5)P_3_ (Xie et al., 2009; Chao et al., 2010), that regulate cellular processes such as signal transduction, vesicle trafficking, actin cytoskeleton dynamics, cell adhesion and cell motility (Loijens and Anderson, 1996; Shulga et al., 2012, 2011).

Cells with an insertion in the *pipkinA* gene (referred to in this report as *pipkinA*^ins^ cells) do not have the ability to develop, fail to form aggregates even after 48 h (Guo et al., 2001) and have a reduced ability to undergo chemotaxis (Guo et al., 2001). During development, pulses of cAMP increase expression of the cAMP receptor and other key proteins needed for aggregation, but this effect does not occur in *pipkinA*^ins^ cells (Guo et al., 2001). In previously published complementation assays, the PI(4)P kinase domain of PIPkinA was replaced with the equivalent sequences from *Saccharomyces cerevisiae* Mss4 (type I), rat type Iβ and human type IIβ PI(4)P 5-kinases (PIP5Ks), and cells expressing these chimeric proteins showed normal development, indicating that *Dictyostelium* PIPkinA is homologous to these other PI(4)P kinases (Guo et al., 2001).

In this report, we made a gene replacement knockout of *pipkinA* and found that PIPkinA is necessary for chemorepulsion from AprA, acting downstream from the AprA receptor to mediate the ability of AprA to inhibit Ras activation. We also find that PIPkinA decreases levels of the phosphatidylinositol phosphates PI(4)P and PI(3,4,5)P_3_ without significantly affecting levels of PI(4,5)P_2_, suggesting the existence of a complex mechanism regulating phosphatidylinositol phosphate levels, and that PIPkinA inhibits cell proliferation and potentiates phagocytosis.

## RESULTS

### PIPkinA regulates *D. discoideum* development and cell size

**Fig. 1.**
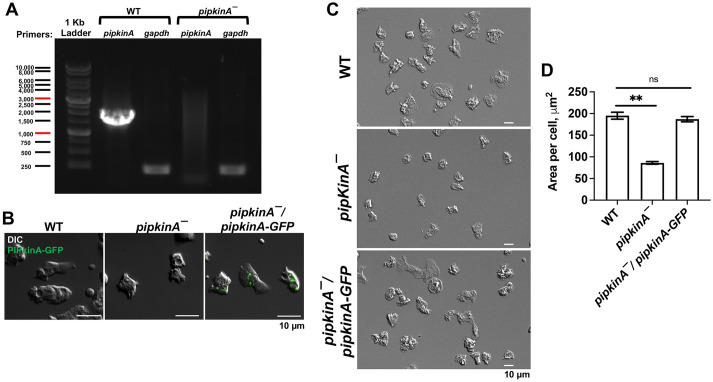
***pipkinA^−^* cells are abnormally small.** (A) Deletion of *pipkinA* was confirmed by PCR using primers for *pipkinA*, with *gapdh* (*gpdA*) used as a loading control. 1 kb ladder band sizes are shown in bp. (B) Differential interference contrast (DIC) and GFP fluorescence images of the indicated strains. Images are representative of three independent experiments. (C) DIC images of the indicated cell strains. Images are representative of three independent experiments. (D) Area per cell for the indicated cell strains. **P<0.01; ns, not significant (unpaired two-tailed *t*-tests, Welch's correction). Values are mean±s.e.m., *n*≥3 independent experiments.

To verify the phenotype of cells lacking PIPkinA, we made a transformant lacking the complete *pipkinA* open reading frame. Blasticidin-resistant clones were screened for homologous recombination by PCR using a primer in the *pipkinA* 5′ flanking region and a primer in the coding region that gave a 1.77 kb product from wild-type (WT) DNA (Fig. 1A; Table S3). Of eight transformant clones screened, four did not show the WT PCR product, and all four of these successful transformants aggregated into mounds but did not form fruiting bodies in the plaques when grown on a bacterial lawn (Fig. S1A,B), unlike the previously described *pipkinA*^ins^ insertion transformant, which does not form aggregates on a *Klebsiella aerogenes* bacterial lawn or on filters after 48 h (Guo et al., 2001). This phenotype difference could be due to differences between the media and/or bacteria used in the Guo et al. (2001) study and our study. Four other transformants had the *pipkinA* PCR product, and these formed aggregates and fruiting bodies in the plaques (data not shown). One of the transformants lacking the *pipkinA* open reading frame was chosen for further analysis and was designated *pipkinA^−^*. We then expressed PIPkinA as a fusion with a green fluorescent protein (GFP) in *pipkinA^−^* cells to make the strain *pipkinA*^−^/*pipkinA-GFP.* Fluorescence microscopy confirmed that, as previously observed (Guo et al., 2001), expression of PIPkinA–GFP caused cells to show fluorescence (Fig. 1B). As previously observed for PIPkinA–GFP expression in *pipkinA^ins^* cells (Guo et al., 2001), expression of the PIPkinA–GFP fusion in *pipkinA^−^* cells rescued the abnormal development of *pipkinA^−^* cells (Fig. S1C). Compared to WT cells, the size of cells lacking PIPkinA was significantly reduced, and this phenotype was also rescued by expression of PIPkinA–GFP (Fig. 1C,D).

### PIPkinA decreases PI(4)P and PI(3,4,5)P_3_ levels

**Fig. 2.**
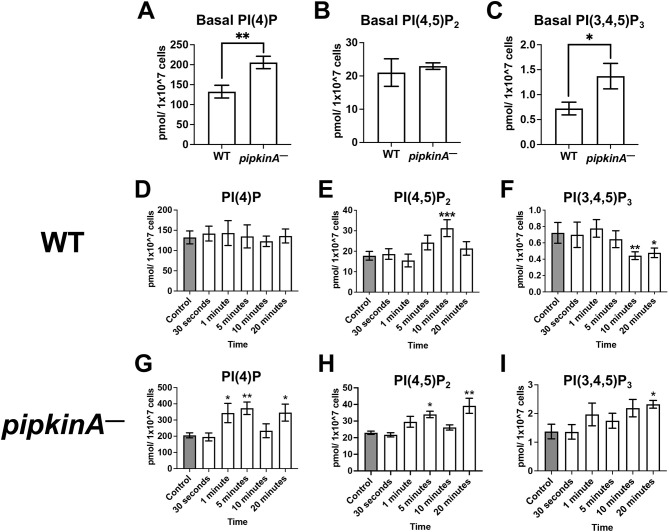
**PIPkinA decreases PI(4)P and PI(3,4,5)P_3_ levels.** WT and *pipkinA^−^* cells were incubated in HL5 and treated with 300 ng/ml of rAprA for the indicated timepoints. Control is exposure to rAprA for 5 s. (A–C) Graphs represent the basal levels of (A) PI(4)P, (B) PI(4,5)P_2_ and (C) PI(3,4,5)P_3_ in WT and *pipkinA^−^* cells without rAprA treatment. (D–F) Graphs represent the levels of (D) PI(4)P, (E) PI(4,5)P_2_ and (F) PI(3,4,5)P_3_ in WT cells. (G–I) Graphs represent the levels of (G) PI(4)P, (H) PI(4,5)P_2_ and (I) PI(3,4,5)P_3_ in *pipkinA^−^* cells. *P<0.05, **P<0.01, ***P<0.001 (compared to the control; unpaired two-tailed *t*-tests, Welch's correction). Values are mean±s.e.m., *n*≥5 independent experiments.

The localizations of PI(4,5)P_2_ and PI(3,4,5)P_3_ at the plasma membrane play a major role in cell migration (Di Paolo and De Camilli, 2006; Huang, 2007). The kinase domain of PIPkinA fused to glutathione-S-transferase expressed in and then purified from bacteria phosphorylates PI(4)P *in vitro* to generate PI(4,5)P_2_ (Guo et al., 2001). To determine the effect of PIPkinA on PI(4)P and PI(4,5)P_2_ levels *in vivo*, PI(4)P, PI(4,5)P_2_ and PI(3,4,5)P_3_ levels were measured in WT and *pipkinA^−^* cells. Compared to WT cells, *pipkinA^−^* cells showed significantly higher basal levels of PI(4)P and PI(3,4,5)P_3_ (Fig. 2A,C). There was no significant difference between the basal levels of PI(4,5)P_2_ in WT cells and *pipkinA^−^* cells (Fig. 2B), indicating that *pipkinA^−^* cells are able to maintain basal levels of PI(4,5)P_2_ through other pathways. The addition of recombinant AprA (rAprA; Bakthavatsalam et al., 2008) to WT cells caused no significant change in PI(4)P levels (Fig. 2D) but increased PI(4,5)P_2_ levels at 10 min and decreased PI(3,4,5)P_3_ levels at 10 and 20 min (Fig. 2E,F). When added to *pipkinA^−^* cells, rAprA also increased PI(4,5)P_2_ levels, albeit with a different time course (Fig. 2H). Unlike in WT cells, rAprA caused an increase in PI(4)P in *pipkinA^−^* cells (Fig. 2G) and increased, rather than decreased, PI(3,4,5)P_3_ levels (Fig. 2I). These data suggest that PIPkinA decreases basal PI(4)P and PI(3,4,5)P_3_ levels, and is necessary for the effect of AprA on PI(4)P and PI(3,4,5)P_3_ levels as well as the timing of the effect of AprA on PI(4,5)P_2_ levels.

### PIPkinA is necessary for chemorepulsion and AprA-induced proliferation inhibition

**Fig. 3.**
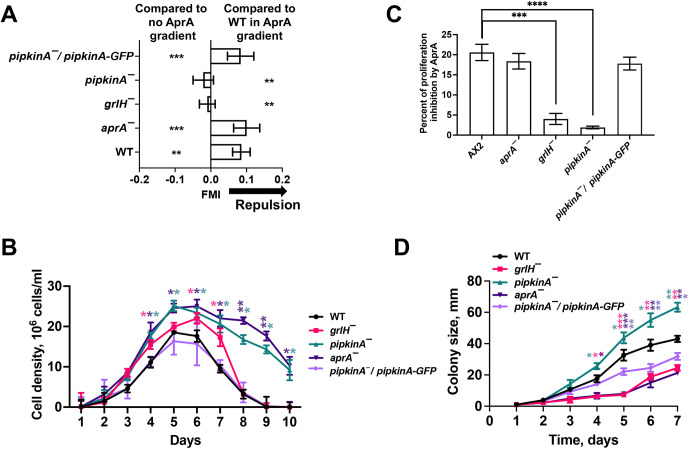
**PIPkinA is required for AprA-induced chemorepulsion, proliferation inhibition and colony expansion.** (A) The indicated strains were imaged for 40 min after 20 min of exposure to HL5 growth medium (control) or to an rAprA gradient in HL5 growth medium in Insall chambers. A positive forward migration index (FMI) indicates chemorepulsion from the AprA, and a negative FMI indicates chemoattraction. The graph shows the FMI for the indicated strains in a rAprA gradient. On the left side of the graph, **P<0.01 and ***P<0.001 compared to the no-gradient control for that genotype. On the right side of the graph, **P<0.01 compared to Ax2 WT in an rAprA gradient. Unpaired two-tailed *t*-tests, Welch's correction. (B) Log-phase cells of the indicated strains were grown in HL5 liquid shaken suspension culture starting at 1×10^5^ cells/ml and counted daily. (C) The indicated strains were cultured in HL5 and either treated with 300 ng/ml rAprA or an equal volume of the AprA buffer (20 mM sodium phosphate, pH 6.9) was added as a control for 16 h. After 16 h, the cell densities were counted. Graph shows the percentage of proliferation inhibition by AprA. (D) For the indicated strains, 10 µl of 1×10^5^ cells/ml cell suspensions were spotted on SM/5 plates with *E. coli* K-12, and the expanding colonies were measured daily. In B–D, *P<0.05, ** *P*<0.01, *** *P*<0.001 and **** *P*<0.0001 compared with Ax2 WT control (unpaired two-tailed *t*-tests, Welch's correction). Values are mean±s.e.m.; *n*≥3 independent experiments.

We have previously identified mutants (such as cells lacking the AprA receptor GrlH) that have defective chemorepulsion from AprA, and some mutants (such as cells lacking AprA) that show chemorepulsion from exogenous AprA (Bakthavatsalam et al., 2009; Rijal et al., 2019; Tang et al., 2018; Kirolos and Gomer, 2022). To determine whether PIPkinA is required for AprA-induced chemorepulsion, cells were exposed to a gradient of rAprA. Unlike WT cells, neither *grlH^−^* cells nor *pipkinA^−^* cells moved away from the source of rAprA (Fig. 3A). Similar to WT cells, *aprA^−^* and *pipkinA^−^*/*pipkinA-GFP* cells were repelled by rAprA (Fig. 3A). As previously observed, rAprA did not significantly affect the speed and persistence of WT, *aprA^−^* or *grlH^−^* cells (Fig. S2A,B) (Rijal et al., 2019), and *pipkinA^−^*and *pipkinA^−^*/*pipkinA-GFP* cells showed normal speed and persistence in the presence or absence of rAprA (Fig. S2A,B). These data indicate that, as suggested by the phenotype of the original *pipkinA* REMI mutant (Kirolos et al., 2022), PIPkinA is necessary for chemorepulsion from AprA. AprA inhibition of cell proliferation requires the G-protein-coupled receptor GrlH and the G protein subunits Gα8 and Gβ (Bakthavatsalam et al., 2009; Phillips and Gomer, 2012). Cells lacking the AprA transduction components GrlH, PakD, QkgA and RblA also exhibit faster proliferation and reach higher cell densities compared to cells of the WT Ax2 strain (Brock and Gomer, 2005; Bakthavatsalam et al., 2009; Phillips and Gomer, 2010; Bakthavatsalam et al., 2014; Phillips and Gomer, 2014; Tang et al., 2018). *aprA*^−^ cells grow faster than Ax2 WT cells in both shaken suspension growth medium cultures and on bacterial lawns (Brock and Gomer, 2005). To determine whether PIPkinA is required for AprA regulation of cell proliferation, cells were grown in HL5 growth medium in shaken suspension culture. *grlH^−^*, *pipkinA^−^* and *aprA^−^* cells showed significantly faster proliferation than WT cells (Fig. 3B; Table S1). As previously observed, *grlH^−^* cells died faster after reaching stationary phase than WT cells (Fig. 3B) (Tang et al., 2018), whereas *pipkinA^−^* and *aprA^−^* cells died slower than WT cells after stationary phase (Fig. 3B). *pipkinA^−^*/*pipkinA-GFP* cells reached a similar cell density at the stationary phase and died at a similar rate as WT cells (Fig. 3B; Table S1). As previously observed (Tang et al., 2018), *grlH^−^* cells showed a decreased sensitivity to AprA-induced proliferation inhibition, whereas *aprA^−^* cells had a response similar to that of WT cells (Fig. 3C). The *pipkinA^−^* cells had a decreased sensitivity to AprA-induced proliferation inhibition, and this was rescued by expression of PIPkinA–GFP (Fig. 3C).

It has previously been shown that *aprA^−^*, *pakD^−^*, *rblA^−^*, *qkgA^−^* and *grlH^−^* cells form abnormally small colonies on lawns of bacteria compared to Ax2 WT cells (Brock and Gomer, 2005; Phillips and Gomer, 2010; Bakthavatsalam et al., 2014; Phillips and Gomer, 2014; Tang et al., 2018; Kirolos and Gomer, 2022). The *pipkinA^−^* cells formed significantly larger colonies compared to WT cells, whereas as previously observed, *aprA^−^* and *grlH^−^* cells formed abnormally small colonies (Fig. 3D; Fig. S3). Interestingly, *pipkinA^−^*/*pipkinA-GFP* cells had reduced colony sizes compared to those of WT cells (Fig. 3D; Fig. S3). Taken together, these results suggest that PIPkinA is required for AprA inhibition of cell proliferation and AprA regulation of colony size, and that for unknown reasons PIPkinA reduces colony size.

**Fig. 4.**
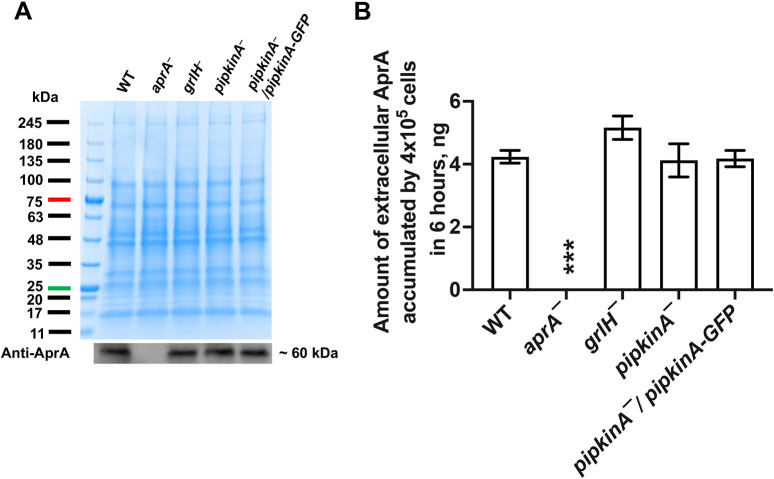
**PIPkinA is not required for extracellular accumulation of AprA.** (A,B) Spots of 4×10^5^ cells of the indicated strains were placed in wells of 8-well slides and allowed to adhere, and 0.2 ml HL5 was gently added to the wells. After 6 h, western blots of the medium in the well were stained for AprA, using known quantities of rAprA as calibration standards. (A) Representative Coomassie-stained gel (top) and anti-AprA western blot (bottom). (B) Quantification of accumulated extracellular AprA. Values are mean±s.e.m. of four independent experiments. ***P<0.001 compared to Ax2 WT (unpaired two-tailed *t*-tests, Welch's correction).

AprA inhibits pseudopod formation at the region of a WT cell closest to a source of AprA (Rijal et al., 2019), and a high uniform concentration (300 ng/ml) of rAprA causes WT cells to round up and decrease their motility speed (Kirolos and Gomer, 2022). To determine whether rAprA affects the roundness and motility of *pipkinA^−^* cells, cells were exposed to a uniform concentration of buffer or 300 ng/ml rAprA and imaged for 60 min. The *pipkinA^−^* cells treated with rAprA did not significantly decrease cell speed (Fig. S2C; Movies 1–4) or increase roundness (Fig. S2D; Movies 1–4) in response to rAprA, and this effect was rescued by expression of PIPkinA–GFP (Fig. S2C,D; Movies 5 and 6). For these assays, cells were in submerged culture exposed to air, whereas for the chemorepulsion assays the cells were in a narrow space in an Insall chamber. The different conditions might account for the slower speed of cells in the Insall chamber compared to that of cells in submerged culture (Fig. S2A,C). The *pipkinA^−^* cell phenotypes described above are not due to a lack of extracellular AprA, as observed for *grlH^−^* cells (Tang et al., 2018; Kirolos and Gomer, 2022); *pipkinA^−^* and *pipkinA^−^*/*pipkinA-GFP* cells showed a normal extracellular accumulation of AprA compared to that of Ax2 WT cells (Fig. 4). These data suggest that PIPkinA is required for rAprA-induced chemorepulsion and proliferation inhibition, and that PIPkinA mediates cell responses to extracellular rAprA.

### PIPkinA is necessary for AprA-induced inhibition of Ras activation

**Fig. 5.**
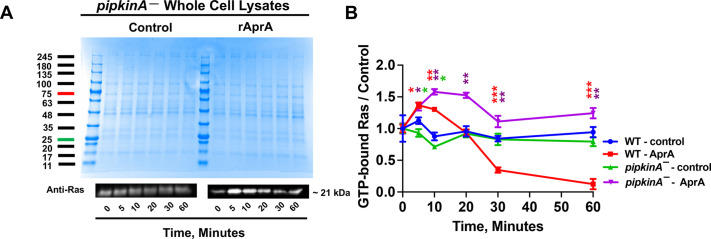
**PIPkinA is necessary for AprA inhibition of Ras activation.** (A) WT and *pipkinA^−^* cells were incubated with or without 300 ng/ml rAprA for the indicated times, and an aliquot of the samples was used for a Coomassie Blue-stained gel to show total protein (top panel, molecular mass indicated in kDa). The remainder of the samples were used for affinity pull-down assays with Raf1-RBD-coated beads. The samples were electrophoresed on SDS–polyacrylamide gels, and western blots of the pull-down samples were stained with an anti-Ras antibody (bottom panel). Band intensities were normalized to the corresponding Coomassie Blue-stained gel scans. Representative data for *pipkinA^−^*cells are shown. The experiments with *pipkinA^−^*cells were performed alongside experiments for WT cells; the WT data are shown in Kirolos and Gomer (2022). (B) Graph shows the levels of GTP-bound Ras for the indicated strains and treatments, normalized to the no-AprA 0 min control for each genotype. Values are mean±s.e.m. of three independent experiments, with the exception of the WT AprA 0, 5, 10 and 20 min data points, which are mean±s.e.m. of five independent experiments. *P<0.05, **P<0.01, ***P<0.001 compared with the 0 min control (unpaired two-tailed *t-*tests, Welch's correction). The WT control data and part of the WT AprA data set are from Kirolos and Gomer (2022).

To inhibit pseudopod formation at the side of a cell closest to a source of AprA and induce chemorepulsion from AprA (Rijal et al., 2019), AprA redistributes Ras cortical activation to the region of the cell away from a source of AprA (Kirolos and Gomer, 2022). GrlH, Gβ, Gα8, PakD, RasG, Erk1 and PKB (also known as PKBA) are necessary for, or are part of, the pathway from the AprA receptor to inhibit Ras cortical activation (Kirolos and Gomer, 2022). To determine whether PIPkinA is necessary for AprA effects on Ras, WT and *pipkinA^−^*, cells were incubated in the presence or absence of rAprA, and then lysed and incubated with beads coated with a GST-tagged Ras-binding domain from Raf1 (Raf1-RBD), which preferentially binds GTP-bound Ras (Rijal et al., 2019). Coomassie-stained gels of the lysates indicated that there were equal amounts of protein in the lysates (Fig. 5A, top panel). Western blots of the lysates stained with anti-Ras antibodies indicated no significant difference in levels of the Ras antigen between WT and *pipkinA^−^* cells. The Raf1-RBD beads were collected by centrifugation and washed, and western blots of the resulting pulldowns of GTP-bound Ras were stained with an antibody that detects Ras (Fig. 5A, lower panel). These assays were performed alongside the assays for WT cells shown in Kirolos and Gomer (2022). As previously described, rAprA treatment decreased levels of GTP-bound Ras after 30 min in WT Ax2 cells (Fig. 5B; Kirolos and Gomer, 2022). Unlike WT cells, in cells lacking PIPkinA, rAprA treatment increased levels of GTP-bound Ras (Fig. 5A,B). Taken together, these results suggest that PIPkinA is necessary for the AprA-induced inhibition of Ras activation.

### PIPkinA potentiates phagocytosis

**Fig. 6.**
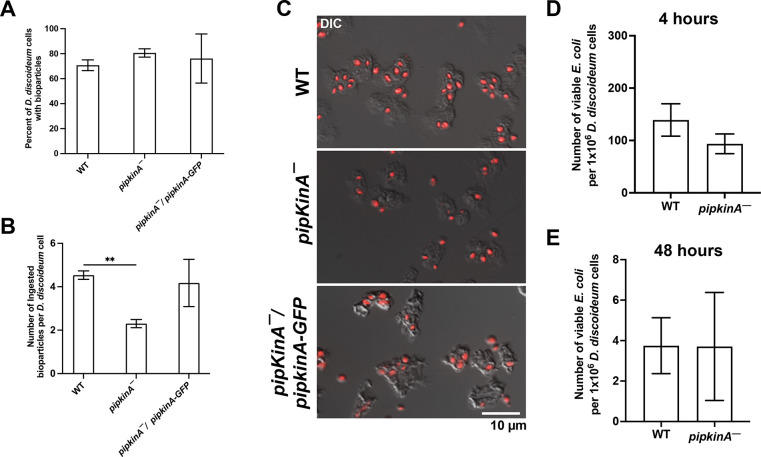
**PIPkinA potentiates phagocytosis.** (A–C) WT, *pipkinA^−^* and *pipkinA^−^*/*pipkinA-GFP* cells were allowed to ingest zymosan A bioparticles for 1 h before being fixed. (A) Graph shows the percentage of cells with ingested bioparticles. (B) Graph shows the number of bioparticles ingested by each cell. (C) Fluorescence and differential interference contrast (DIC) images of fixed cells. Zymosan A bioparticles are shown in red. Images are representative of three independent experiments. (D,E) WT and *pipkinA^−^* cells were incubated with *E. coli* for 2 h then treated with gentamicin for 2 h to kill extracellular bacteria. The cells were then lysed at (D) 4 h and (E) 48 h, and lysates were spread on LB agar plates before being incubated overnight at 37°C. Graphs show viable intracellular *E. coli* per 10^6^ *D. discoideum* cells. **P<0.01 (unpaired two-tailed *t*-tests, Welch's correction). Graphs show mean±s.e.m., *n*≥3 independent experiments.

The conversion of PI(4,5)P_2_ to PI(3,4,5)P_3_ is fundamental for vesicular trafficking (Posor et al., 2013). The localization of PI(4,5)P_2_ in different compartments of the cell surface is directly correlated to intracellular trafficking, such as endocytosis and exocytosis (Schauer and Goud, 2014). PI(4,5)P_2_ interacts with proteins to control the formation and spatiotemporal organization of complexes involved in intracellular trafficking (Sun et al., 2018) and plays a role in clathrin-mediated endocytosis (Jost et al., 1998). To determine whether PIPkinA is required for phagocytosis, WT, *pipkinA^−^* and *pipkinA^−^*/*pipkinA-GFP* cells were allowed to ingest zymosan A bioparticles. The loss of PIPkinA had no significant effect on the percentage of cells with ingested bioparticles (Fig. 6A,C) but decreased the average number of ingested bioparticles per cell (Fig. 6B,C). We have previously found that after 48 h, some *E. coli* bacteria ingested by *D. discoideum* cells remain viable (Rijal et al., 2020). To determine whether PIPkinA plays a role in bacterial survival, cells were incubated with *E. coli* K-12 for 2 h to allow ingestion of bacteria. After 2 h, the wells were washed, and gentamicin was added. Gentamicin is an antibiotic that cannot be ingested by eukaryotes and only kills uningested bacteria (Ronn et al., 2017; VanCleave et al., 2017). The loss of PIPkinA had no significant effect on the number of viable ingested *E. coli* at 4 h and 48 h (Fig. 6D,E). These data indicate that PIPkinA slightly potentiates phagocytosis but has no discernible effect on the killing of ingested bacteria.

### PIPkinA does not significantly affect AprA binding to cells

**Fig. 7.**
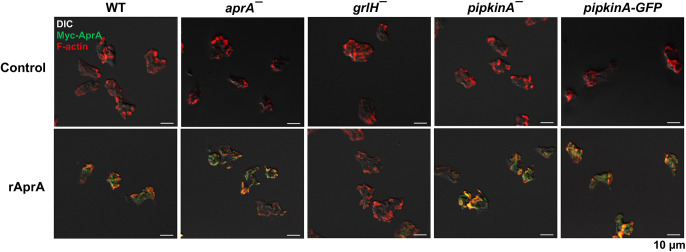
**Myc-tagged rAprA binding to cells.** Cells were incubated in the absence or presence of 300 ng/ml Myc-tagged rAprA for 10 min and then fixed and stained with a rabbit anti-Myc tag antibody to detect Myc–AprA (green) and with phalloidin-iFluor 555 to detect F-actin (red). Combined differential interference contrast (DIC) and fluorescence images are shown. Images are representative of three independent experiments.

rAprA binds to GrlH, and cells lacking GrlH show very little binding of rAprA (Tang et al., 2018). To determine whether the loss of PIPkinA affects AprA binding to cells, cells were incubated with Myc-tagged rAprA, washed and stained for the Myc tag using a rabbit anti-Myc antibody (Fig. 7, green). The tagged AprA bound to *aprA^−^*, *pipkinA^−^* and *pipkinA^−^*/*pipkinA-GFP* cells but showed reduced binding to *grlH^−^* cells (Fig. 7; Fig. S4A). Western blots of cells similarly treated, and then mechanically lysed and separated by centrifugation at 14,000 ***g*** for 60 min into pellet and supernatant fractions, were stained for Myc (rAprA) and for actin as a loading control (Figs S4B,S5). rAprA was observed in the pellet fraction, which contains plasma membranes (Rijal et al., 2022; Ostrom and Insel, 2006), of WT, *aprA^−^*, *pipkinA^−^* and *pipkinA^−^*/*pipkinA-GFP* cells (Figs S4B,S5). *grlH^−^* cells had little to no rAprA bound to the pellet (Figs S4B,S5). These data suggest that PIPkinA does not affect AprA binding to cells and plays a role downstream of the AprA receptor.

## DISCUSSION

**Fig. 8.**
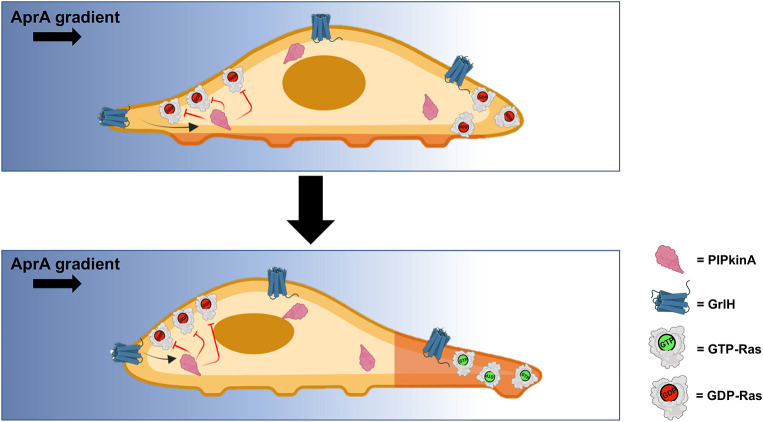
**Proposed mechanism for PIPkinA involvement in chemorepulsion.** Top: in an AprA gradient, the AprA receptor GrlH is activated by high concentrations of AprA, and through the involvement of PIPkinA, inhibits Ras activation (keeps Ras in the GDP-bound configuration) at the side of a cell closest to the source of AprA. Because Ras activation is needed to form a pseudopod, there is little or no pseudopod formation at the side of the cell closest to the source of AprA. At the right side of the figure, Ras happens to be in an inactive state, and a pseudopod is not forming at this location. Bottom: at the right side of the figure, Ras now activates, and a pseudopod forms. The Ras activation (GTP-bound Ras)-mediated random formation of pseudopods allows cells to form pseudopods and move in essentially any direction except toward the source of AprA, thus causing chemorepulsion from AprA.

A previous genetic screen using REMI suggested that PIPkinA is involved in *D. discoideum* chemorepulsion from AprA (Kirolos et al., 2022), and here we showed that PIPkinA is indeed necessary for AprA-induced chemorepulsion, further supporting the usefulness of the REMI mutagenesis approach (Kuspa and Loomis, 1992). PIPkinA is also necessary for AprA-induced inhibition of cell proliferation. Possibly due to a high proliferation rate, *pipkinA^−^* cells are abnormally small. PIPkinA mediates the AprA effects on chemorepulsion and proliferation without significantly affecting the extracellular accumulation of AprA or binding of AprA to cells, suggesting that PIPkinA acts downstream from the AprA receptor (Fig. 8).

AprA induces chemorepulsion by inhibiting Ras activation at the side of the cell closest to the source of AprA (Kirolos and Gomer, 2022), and this in turn inhibits formation of pseudopods at the side of the cell closest to the source of AprA to bias random pseudopod formation (Rijal et al., 2019). In both mammalian and *D. discoideum* cells, activation of Ras can also potentiate cell proliferation (Coleman et al., 2004; Sutherland et al., 2001). PIPkinA is necessary for AprA inhibition of Ras activation. This suggests that AprA activation of the AprA receptor uses a pathway involving PIPkinA to inhibit Ras activation to both induce chemorepulsion and inhibit proliferation (Fig. 8).

PIPkinA converts PI(4)P to PI(4,5)P_2_ (Guo et al., 2001). Although the mechanism is unclear, PI(4,5)P_2_ can affect Ras subcellular localization and Ras activity (Cao et al., 2019; Heo et al., 2006; Gulyas et al., 2017). Conversely, Ras can activate phospholipase C-β and -γ to break down PI(4,5)P_2_ (Foster and Xu, 2003; Snyder et al., 2003; Walliser et al., 2008; El-Sibai and Backer, 2007; Gresset et al., 2012). Active Ras mediates the recruitment and activation of PI3K (Castellano and Downward, 2010) to phosphorylate PI(4,5)P_2_ to PI(3,4,5)P_3_, and PI(3,4,5)P_3_ can enhance the activity of Ras (Sasaki et al., 2004, 2007). By regulating the levels of phosphatidylinositol phosphates, it is thus conceivable that PIPkinA could mediate AprA inhibition of Ras activation.

In cells lacking PIPkinA, we would expect a high basal level of PI(4)P, low basal level of PI(4,5)P_2_ and low basal level of PI(3,4,5)P_3_. As we expected, we observed that cells lacking PIPkinA had a high basal level of PI(4)P. However, *pipkinA^−^* cells showed no change in the basal level of PI(4,5)P_2_ and a high basal level of PI(3,4,5)P_3_. This suggests that additional pathways not involving PIPkinA regulate basal PI(4,5)P_2_ and PI(3,4,5)P_3_ levels.

In mammalian cells, phosphorylation of PI(4,5)P_2_ to generate PI(3,4,5)P_3_, and activation of Ras, can induce phagocytosis (Desale and Chinnathambi, 2021; Buckley et al., 2020; Marshall et al., 2001; Vieira et al., 2001; Araki et al., 2007). In *D. discoideum*, localized activation of Ras and the accumulation of PI(3,4,5)P_3_ also generate a phagocytic cup (Veltman et al., 2016), and RasS-null cells show impaired phagocytosis (Chubb et al., 2000). In mammalian cells, PIP5K-mediated generation of PI(4,5)P_2_ potentiates phagocytosis (van den Bout and Divecha, 2009). Since PIPkinA appears to inhibit Ras activation but potentiates phagocytosis, some inhibition of Ras activation, possibly at regions outside the phagocytic cup, might be needed for efficient phagocytosis. Alternatively, PIPkinA might potentiate phagocytosis directly by generating PI(4,5)P_2_ similar to PIP5K, or indirectly by decreasing basal levels of PI(3,4,5)P_3_.

In WT cells, AprA caused no significant change in PI(4)P levels, whereas in *pipkinA^−^* cells, AprA increased PI(4)P levels. This suggests that PIPkinA counteracts some AprA-induced activity that increases PI(4)P levels. In both WT and *pipkinA^−^* cells, AprA increased PI(4,5)P_2_ levels, albeit with different time courses. This suggests that AprA might increase CnrN and PTEN activity to convert PI(3,4,5)P_3_ to PI(4,5)P_2_ (Tang and Gomer, 2008a,b), and that PIPkinA might also be generating PI(4,5)P_2_ to affect the time course of the AprA-induced increase in PI(4,5)P_2_. The regulation of PI(4,5)P_2_ and PI(3,4,5)P_3_ levels involve complex pathways (Huang et al., 2003; Buczynski et al., 1997; Zhou et al., 1998; Loovers et al., 2006; van Haastert et al., 2007). Since the basal levels of PI(3,4,5)P_3_ are lower than both the basal and AprA-increased levels of PI(4,5)P_2_, other pathways are likely to be involved in the AprA-induced increase in PI(4,5)P_2_ levels. In WT cells, AprA decreased PI(3,4,5)P_3_ levels, whereas in *pipkinA^−^* cells, AprA increased PI(3,4,5)P_3_ levels. PIPkinA inhibits Ras activation, and Ras activates PI3K (Castellano and Downward, 2010; Vivanco and Sawyers, 2002). The resulting AprA-induced inhibition of PI3K, combined with the known AprA-induced increase in PTEN activity, could explain the AprA-induced decrease in PI(3,4,5)P_3_ levels in WT cells. The observed increase in PI(3,4,5)P_3_ levels in *pipkinA^−^* cells suggests that AprA activates an additional pathway that, in the absence of PIPkinA, increases PI(3,4,5)P_3_ levels. Taken together, these observations suggest that PIPkinA is part of a complex network that regulates levels of phosphatidylinositol phosphates, and that PIPkinA mediates chemorepulsion and inhibits proliferation by inhibiting Ras.

## MATERIALS AND METHODS

### Cell strains and the generation of *pipkinA^−^* cells

*D. discoideum* strains were purchased from the *Dictyostelium* stock center (Fey et al., 2013). The strains included Ax2, *grlH^−^* (DBS0350226) (Tang et al., 2018) and *aprA^−^* (DBS0235509) (Brock and Gomer, 2005). *pipkinA^−^* cells were generated by homologous recombination in the Ax2 background using the vector pLPBLP, which has two *loxP* sites flanking both sides of the Bsr expression cassette *act15/Bsr* (Faix et al., 2004). The 5′ flanking region (542 bp) and the 3′ flanking region (542 bp) of *pipkinA* were amplified by PCR from genomic DNA extracted from Ax2 vegetative cells and directionally cloned into pLPBLP. Primers used to amplify these regions for the PIPkinA gene are listed in Table S2. The resulting construct, with 4767 bp of the 4767 bp *pipkinA* coding region replaced with a blasticidin resistance construct, was linearized with KpnI (NEB, Ipswich, MA, USA) and then electroporated into WT Ax2 cells (Gaudet et al., 2007). After 10 days, there were a few visible colonies in the *pipkinA^−^* transformed plate, but none in the control. Cells from the entire plate were collected and seeded on lawns of *E. coli* K-12 on an SM/5 (2 g/l glucose, 2 g/l bacto peptone, 0.2 g/l yeast extract, 0.2 g/l MgSO_4_·7H_2_O, 1.9 g/l KH_2_PO_4_, 1 g/l K_2_HPO_4_, 15 g/l agar) plate for single clone isolation. After 4 days, cells in plaques on the lawns were picked and cultured in HL5 (HL5 with glucose; Formedium, Hunstanton, UK) with 10 µg/ml blasticidin. The genomic DNA from each clone was extracted and purified using a Quick DNA Miniprep kit (ZYMO Research, Irvine, CA, USA), then underwent PCR to verify the correct orientation of the insert using the primers listed in Table S3.

### Generation of *pipkinA^−^/pipkinA-GFP* cells

*pipkinA^−^* cells were transformed with the *D. discoideum* plasmid PKG3016 (a gift from Dr Catherine Pears, Department of Biochemistry, University of Oxford, UK) by electroporation (Rijal et al., 2019; Kuspa and Loomis, 1992). This plasmid contains an actin15 (*act15*) promoter driving the expression of the *pipkinA* cDNA open reading frame (encoding residues 31 to 4767) fused in frame to a C-terminal GFP tag (Guo et al., 2001). The plasmid was sequenced to confirm the construct and that the GFP was in frame. Cells were cultured as previously described with 10 µg/ml G418 in HL5 (Rijal et al., 2019). Images in Fig. 1B,C were taken with differential interference contrast (DIC) and fluorescence optics on a Nikon Ti2 microscope with a 40× objective.

### Images of aggregates

Images of fruiting bodies and aggregates were performed as described previously (Brock et al., 2002) with the exception that a 3.1MP Aptina color CMOS camera (AmScope, Irvine, CA, USA) was mounted on a Microphot-FX microscope (Nikon, Tokyo, Japan). A 2× objective was used, and the end of the lens was covered with aluminum foil with a 2 mm diameter pinhole to increase depth of field.

### PI(4)P, PI(4,5)P_2_ and PI(3,4,5)P_3_ extraction and ELISAs

For the phosphatidylinositol extractions, 1.25×10^7^ cells per test sample were resuspended in fresh HL5 medium and then stimulated with 300 ng/ml rAprA for the indicated times, with the control at 5 seconds of rAprA exposure. The reaction was stopped with an equal volume of ice cold 1 M trichloroacetic acid (TCA) and incubated on ice for 5 min. Phosphatidylinositol extractions and ELISAs were performed using phosphatidylinositol 4-phosphate [PI(4)P] Mass ELISA kits (#K-4000E; Echelon Biosciences, Salt Lake City, UT, USA), phosphatidylinositol 4,5-bisphosphate [PI(4,5)P_2_] Mass ELISA kits (#K-4500, Echelon Biosciences) and phosphatidylinositol 3,4,5-trisphosphate [PI(3,4,5)P_3_] Mass ELISA kits (#K-2500S, Echelon Biosciences). Vacuum-dried samples were stored at −20°C and analyzed by ELISA within 6 months of collection. Test samples were reconstituted with PBS-Tween-20/0.25% Protein Stabilizer solution, included with the kit, and assayed in duplicate.

### Chemorepulsion, proliferation and phagocytosis assays

Recombinant AprA (rAprA) was expressed and purified as previously described (Bakthavatsalam et al., 2008), then concentrated and stored as described in Kirolos and Gomer (2022). Chemorepulsion assays using an Insall chamber (Muinonen-Martin et al., 2010), which allows visualization of cell migration in the presence of a stimulus gradient, were performed as previously described (Bakthavatsalam et al., 2008; Rijal et al., 2019). Each batch of rAprA was tested for chemorepulsion activity on WT cells before further experiments were performed. For each individual experiment, at least 40 cells were tracked. Proliferation in HL5 shaken suspension culture and AprA cell proliferation inhibition assays were performed as previously described (Herlihy et al., 2013; Tang et al., 2018). Colony expansion assays were performed as previously described (Tang et al., 2018). *E. coli* K-12 survival in *D. discoideum* and phagocytosis of Alexa Fluor 594-conjugated zymosan A bioparticles (Invitrogen, Eugene, OR, USA) by *D. discoideum* cells were assayed as described previously (Rijal et al., 2020).

### AprA binding assay

*D. discoideum* cells were grown to 1.0×10^6^ cells/ml in HL5 in shaken suspension culture (160 rpm) and collected by centrifugation at 500 ***g*** for 3 min. Cells were resuspended in HL5 and washed twice more by centrifugation and resuspension. The cells were then resuspended in HL5 to 1.0×10^5^ cells/ml, and 300 µl of the cell suspension was placed in a chamber of a type 354118 8-chamber tissue culture treated glass slide (Corning, Corning, NY, USA). Cells were then allowed to adhere for 30 min at room temperature. From a 250–300 ng/µl Myc-tagged rAprA stock in 20 mM NaPO_4_ pH 7.4, ∼0.5–0.8 µl was added gently to each well to produce a uniform concentration as described previously (Kirolos and Gomer, 2022). Control wells had an equal volume of 20 mM NaPO_4_ pH 7.4 uniformly added to each well. The slides were incubated for 10 min in a humid chamber, then fixed and stained as described previously (Rijal et al., 2019), using a 1:500 dilution of rabbit anti-Myc tag antibody (#71D10; Cell Signaling Technology, Danvers, MA, USA) and1 µg/ml Alexa Fluor 488 donkey anti-rabbit IgG secondary antibody (#711-546-152; Jackson ImmunoResearch, West Grove, PA, USA). Fluorescence labeling of F-actin was done with Phalloidin–iFluor 555 (Abcam, Waltham, MA, USA) following the manufacturer's directions. Images were taken on a Nikon Ti2 Eclipse microscope with a 40× objective. FIJI ImageJ software (Schindelin et al., 2012) was used to analyze and quantify the staining. For each individual experiment, AprA binding to the cell membrane was assessed in at least 35 randomly chosen cells in each well. To measure rAprA protein levels in cell fractions, cells were grown, collected and washed as described above. The cells were then resuspended to 1.0×10^6^ cells/ml in HL5 with 1 ml/well in a 24 well plate (#353047, Corning) and allowed to adhere for 30 min. rAprA was added to a final concentration of 300 ng/ml, or an equal volume of sodium phosphate buffer was added. Cells were lysed and fractionated by centrifugation at 14,000 ***g*** for 1 h at 4°C into supernatant and pellet as described previously (Ostrom and Insel, 2006). Samples were electrophoresed and blotted as described in Kirolos and Gomer (2022) and stained as previously described (Rijal et al., 2019) with 1:250 rabbit anti-Myc tag antibody (#71D10; Cell Signaling Technology), and the secondary antibody was 250 ng/ml peroxidase-conjugated donkey anti-rabbit IgG (#711-036-152, Jackson ImmunoResearch). Staining was detected with SuperSignal West Pico PLUS Chemiluminescent Substrate for 10 min (Thermo Fisher Scientific, Waltham, MA, USA). Images of the membrane were taken using a ChemiDoc XRS system (Bio-Rad, Hercules, CA, USA) and quantified using Image Lab software (Bio-Rad).

### Extracellular AprA concentrations and Ras pulldown assays

Extracellular AprA concentrations were measured as described previously (Brock and Gomer, 2005), using the rabbit anti-AprA antibody, dilutions and methods described in that publication. Ras pulldown assays were performed as described in Kirolos and Gomer (2022), using a Ras Pull-down Activation Assay kit (BK008; Cytoskeleton, Denver, CO, USA).

### Statistics

Prism 8.4.1 (GraphPad, San Diego, CA, USA) was used for *t-*tests and one-way or two-way ANOVA with appropriate post-tests. Significance was defined as *P*<0.05.

## Supplementary Material

10.1242/joces.260541_sup1 Supplementary information
