# Supplementary material for: A phosphatidylinositol phosphate kinase inhibits Ras activation and regulates chemorepulsion in Dictyostelium discoideum
Source: J Cell Sci. 2023 Jul 27;136(14):jcs260541. doi: 10.1242/jcs.260541 (PMC10399982; doi:10.1242/jcs.260541)
Supplement: Supplementary information [file joces-136-260541-s1.pdf]

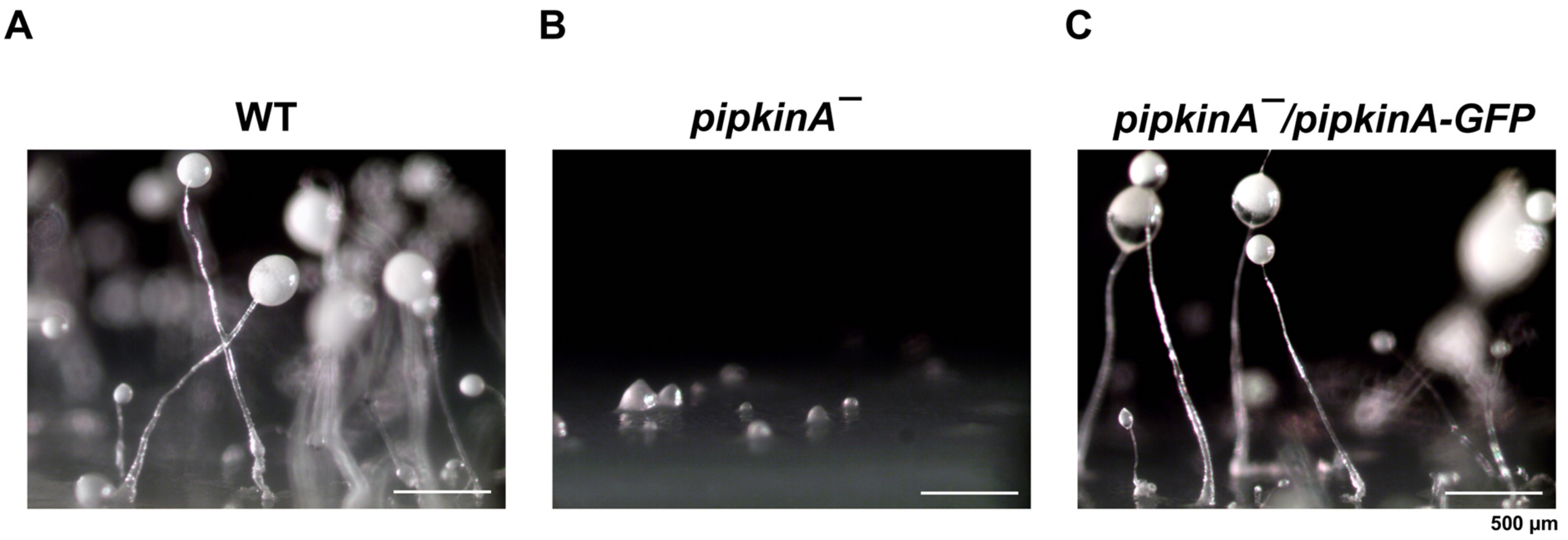


**Fig. S1. Disruption of pipkinA inhibits development.** Cells were allowed to grow on lawns of bacteria, and then overgrow the bacteria and develop. Bars are 500 µm. Images are representative of 3 independent experiments.

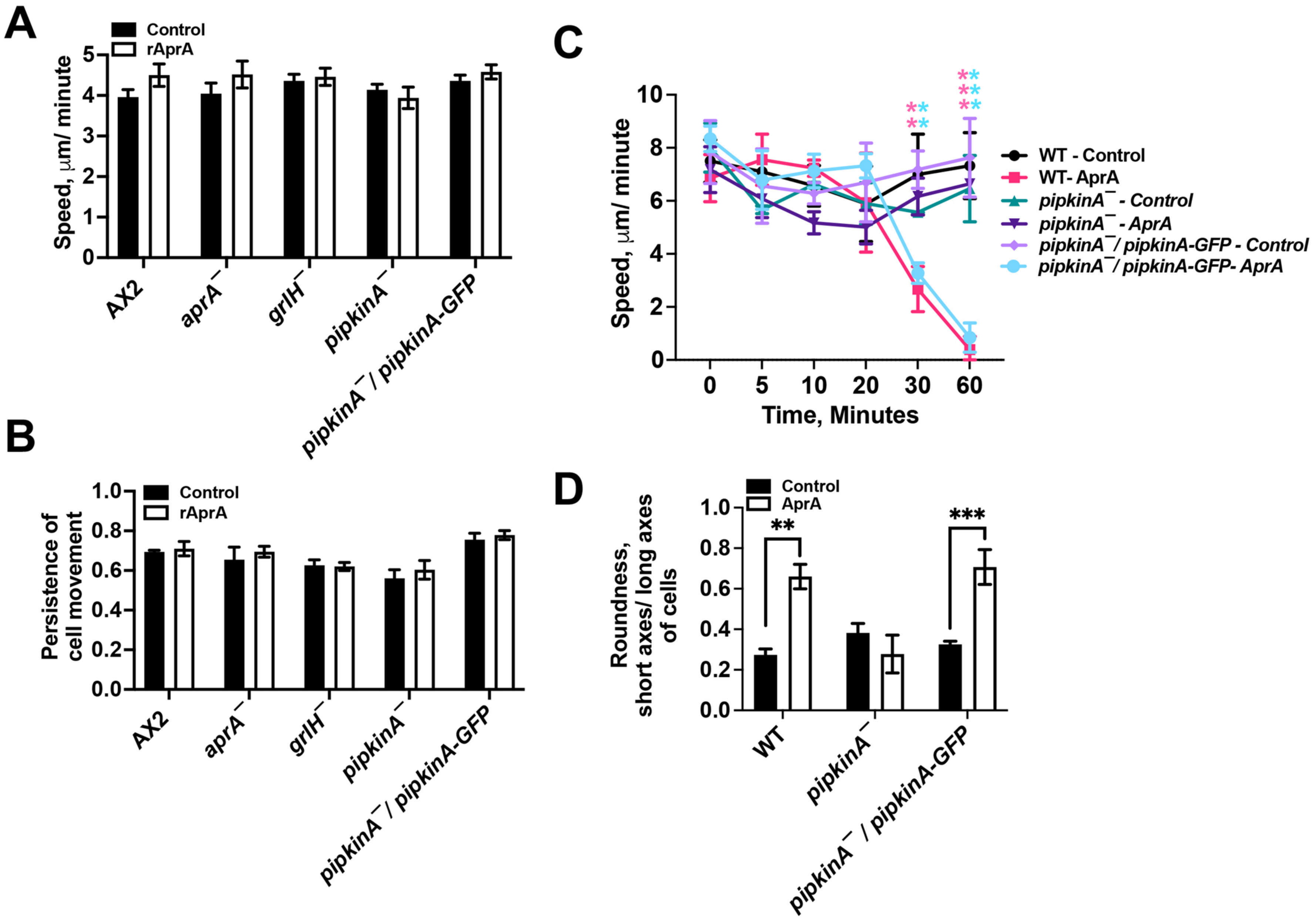


**Fig. S2. AprA does not significantly affect the speed or roundness of *pipkinA*⁻ cells.** (**A**) Graph shows the speed of the indicated strains in the Figure 3 chemorepulsion assays. (**B**) Graph shows the persistence of cell movement in the Figure 3 chemorepulsion assays. **(C)** Graph shows speed of cells in submerged culture in the presence or absence of rAprA. ** indicates $p < 0.01$ and *** $p < 0.001$, compared to Ax2 WT (Unpaired two-tailed t-tests, Welch's correction). **(D)** Roundness of cells exposed to rAprA for 60 minutes was determined by measuring the ratio of the short and long axes of the cell (short/long). ** indicates $p < 0.01$ and *** $p < 0.001$ (2-way ANOVA, Holm-Šídák's test). For A and B, values are mean ± SEM, $n \geq 3$ independent experiments. For C and D, values are mean ± SEM of the averages of 4 independent experiments with at least 45 randomly chosen cells examined for each condition in each experiment.

Journal of Cell Science • Supplementary information

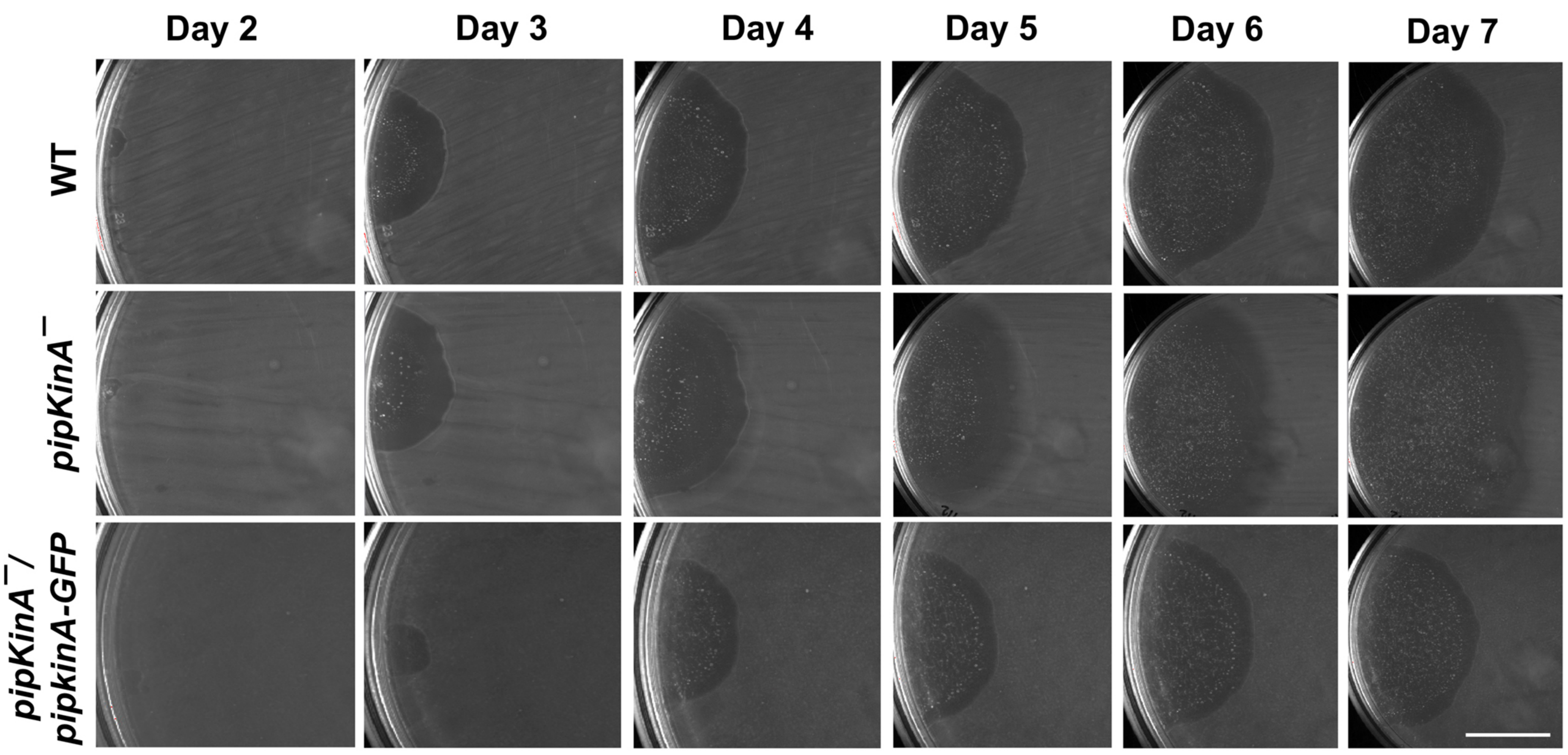


**Fig. S3. *pipkinA*⁻ cells show faster colony expansion on a bacterial lawn.** Representative images of the data graphed in Figure 3D. A spot of $10^5$ cells was placed at the left edge of the plate on day 0. Bar is 20 mm.

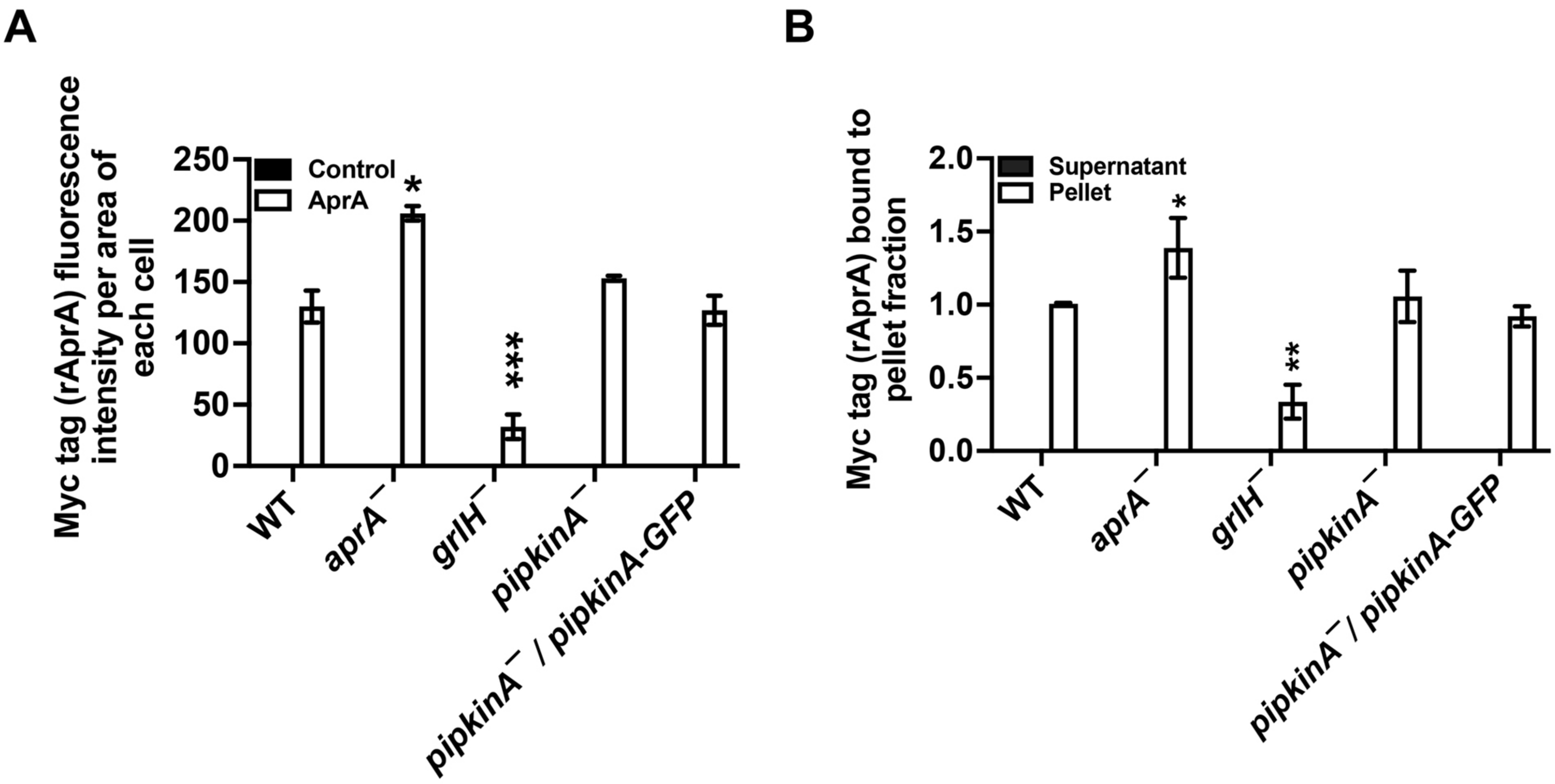


**Fig. S4. Loss of PIPkinA does not affect rAprA binding to cells.** (**A**) Using the images from Figure 7, graph shows Myc-rAprA fluorescence per area of each cell. * indicates $p < 0.05$ and *** $p < 0.001$ compared to Ax2 WT (Unpaired two-tailed t-tests, Welch's correction). Values are mean ± SEM of the averages from 3 independent experiments, with at least 40 randomly chosen cells examined for each point in each experiment. (**B**) Graph shows Myc-rAprA bound to the supernatant and pellet fraction from the experiments in Supplemental Figure 5. * indicates $p < 0.05$ and ** $p < 0.01$ compared to the WT pellet control (Unpaired two-tailed t-tests, Welch's correction). Values are mean ± SEM from 3 independent experiments.

Journal of Cell Science • Supplementary information

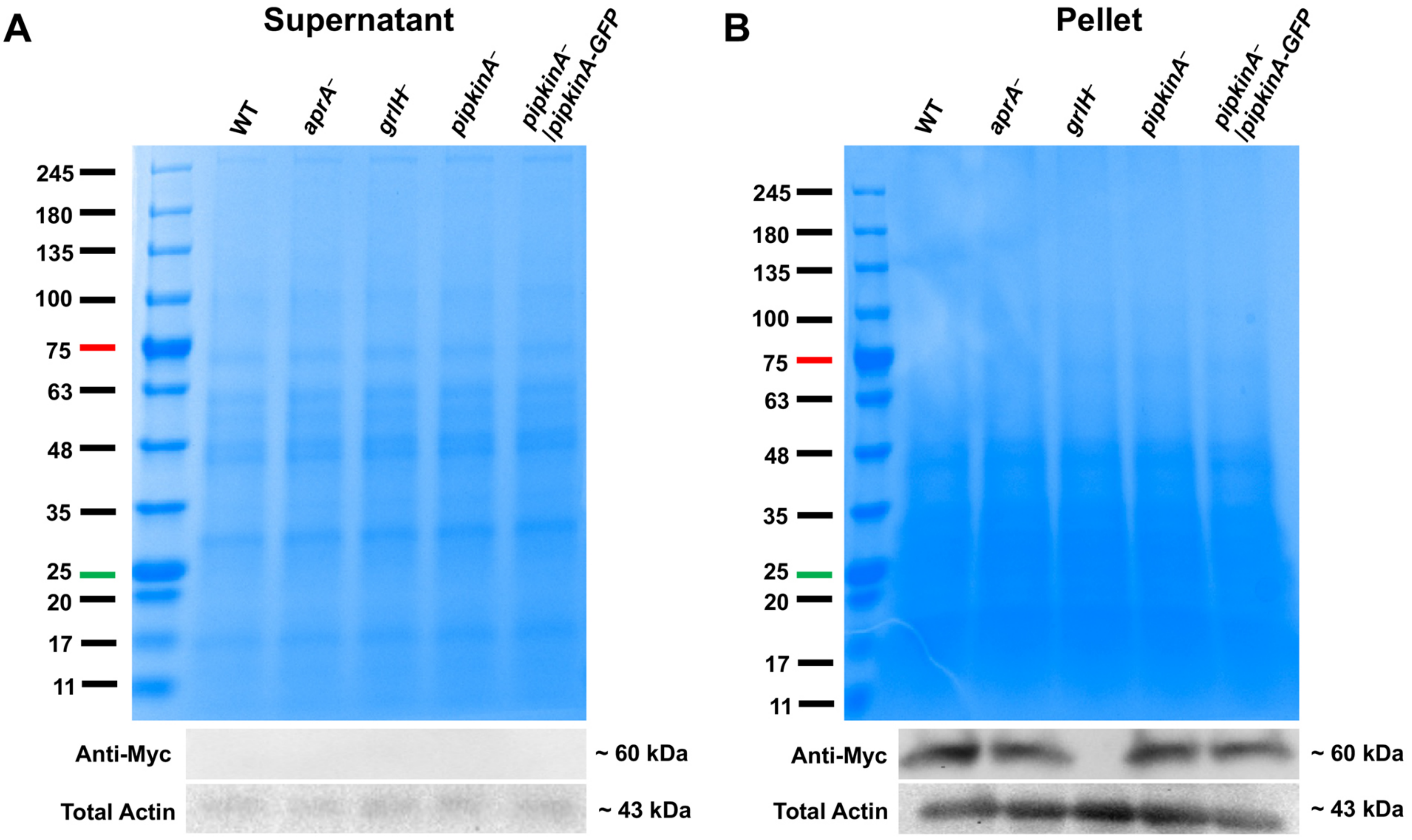


**Fig. S5. Extracellular rAprA binds to the cell membrane.** Cells were incubated with buffer or 300 ng/ml of myc-tagged rAprA for 10 minutes then lysed and fractioned. Western blots of the **(A)** supernatant and the **(B)** pellet were stained with anti-Myc antibodies, and anti-actin antibodies as the loading control.

**Table S1. Doubling times and maximum cell densities. Related to Figure 3.**

| Cell type | Doubling time (h) | Maximum observed cell density ($10^6$ cells/ml) |
|---|---|---|
| WT | 15.1±0.3 | 19.2±1.2 |
| *grlH*$^-$ | 13.9±0.4* | 21.5±1.1 |
| *pipkinA*$^-$ | 13.1±0.5$^‡$ | 24.3±0.9* |
| *aprA*$^-$ | 13.2±0.2$^‡$ | 24.5±1.4* |
| *pipkinA*$^-$*/pipkinA-GFP* | 14.6±0.7 | 15.8±2.1* |

Values are mean±s.e.m., $n$≥3 independent experiments. *$P$<0.05; $^‡P$<0.01 compared to WT (unpaired two-tailed $t$-tests, Welch's correction).

Journal of Cell Science • Supplementary information

**Table S2. Primers used for gene disruptions. Underlined nucleotides indicate restriction sites.**

| **Primer name** | **Primer sequence (5’-3’)[a]** |
|---|---|
| ***Upstream-pipkinA-F-KpnI*** | CGCGCCGGTACCATGACAATATGTCAACCAATTCCATGTGG |
| ***Upstream-pipkinA-R-HindIII*** | CGCCGAAGCTTGCCACGATTTTTCTTTTTTTAATAATTTTAATTTCTTCATTATCTCTAC |
| ***Downstream-pipkinA-F-NdeI*** | CGCGGCATATGGGCTCTGTCATCAATAGAAAAGTTGACAAG |
| ***Downstream-pipkinA-R-NotI*** | GGCCGCGGCCGCTTATTGAACAATTTTTGAAACATAATGTTGAAATCTTTTTCG |

**Table S3. Primers used for verifying gene disruption.** Primers used for verifying complete knockout of the PipKinA gene.

| Primer name | Primer sequence (5’-3’) |
|---|---|
| PipKinA-KO-R | CAGGTATACCACAAGTATTTGTTAATG |
| 5’ region_PipKinA_F | CATTTATTTTTTTACCAACCC |

Journal of Cell Science • Supplementary information

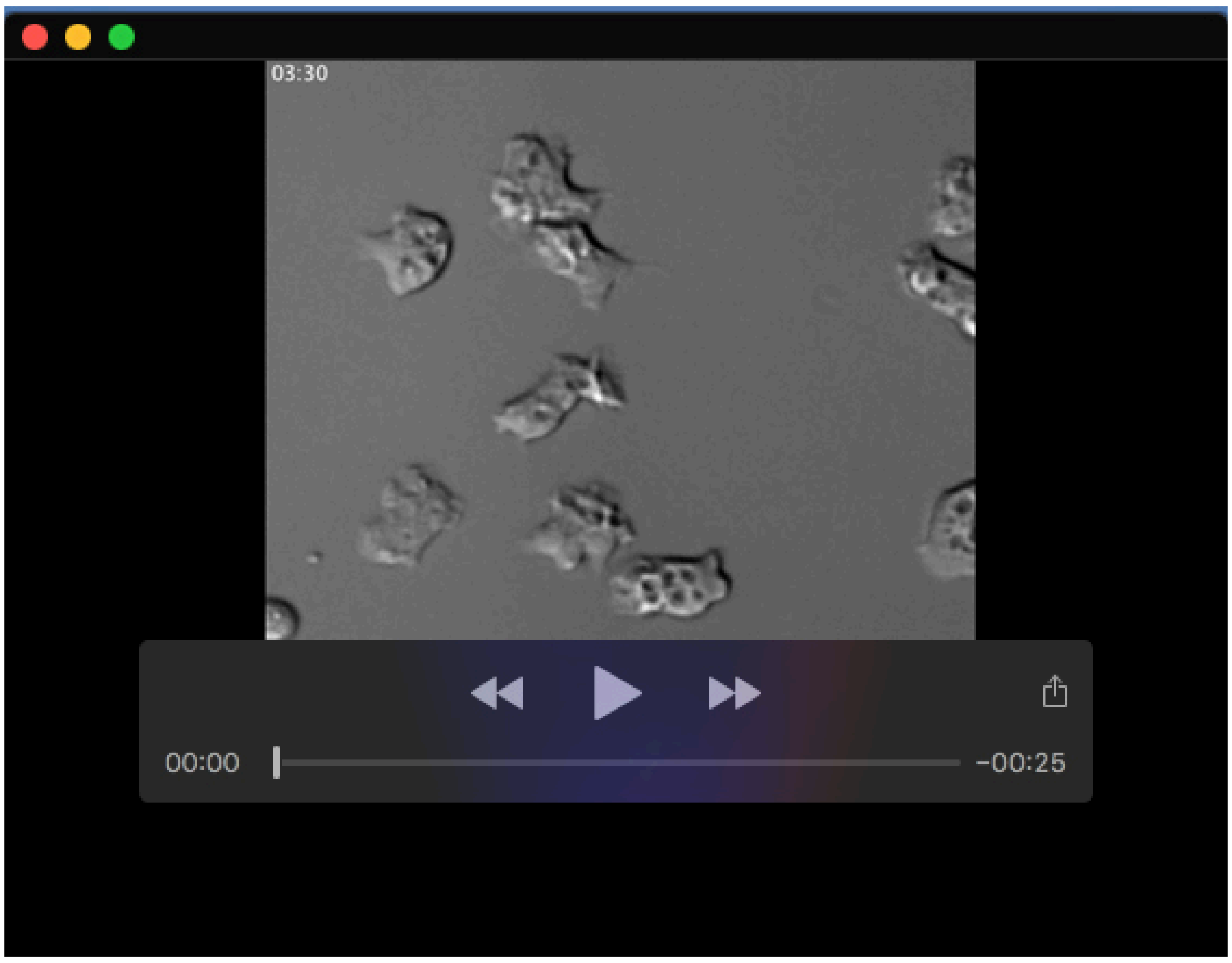


**Movie 1. Motility of WT cells in the absence of exogenous AprA.** Ax2 cells were placed in wells in HL5 and were allowed to adhere. After 30 minutes, cells were imaged. Elapsed time in minutes:seconds is at upper left. The horizontal field of view is 107 µm. Video is representative of 4 independent experiments.

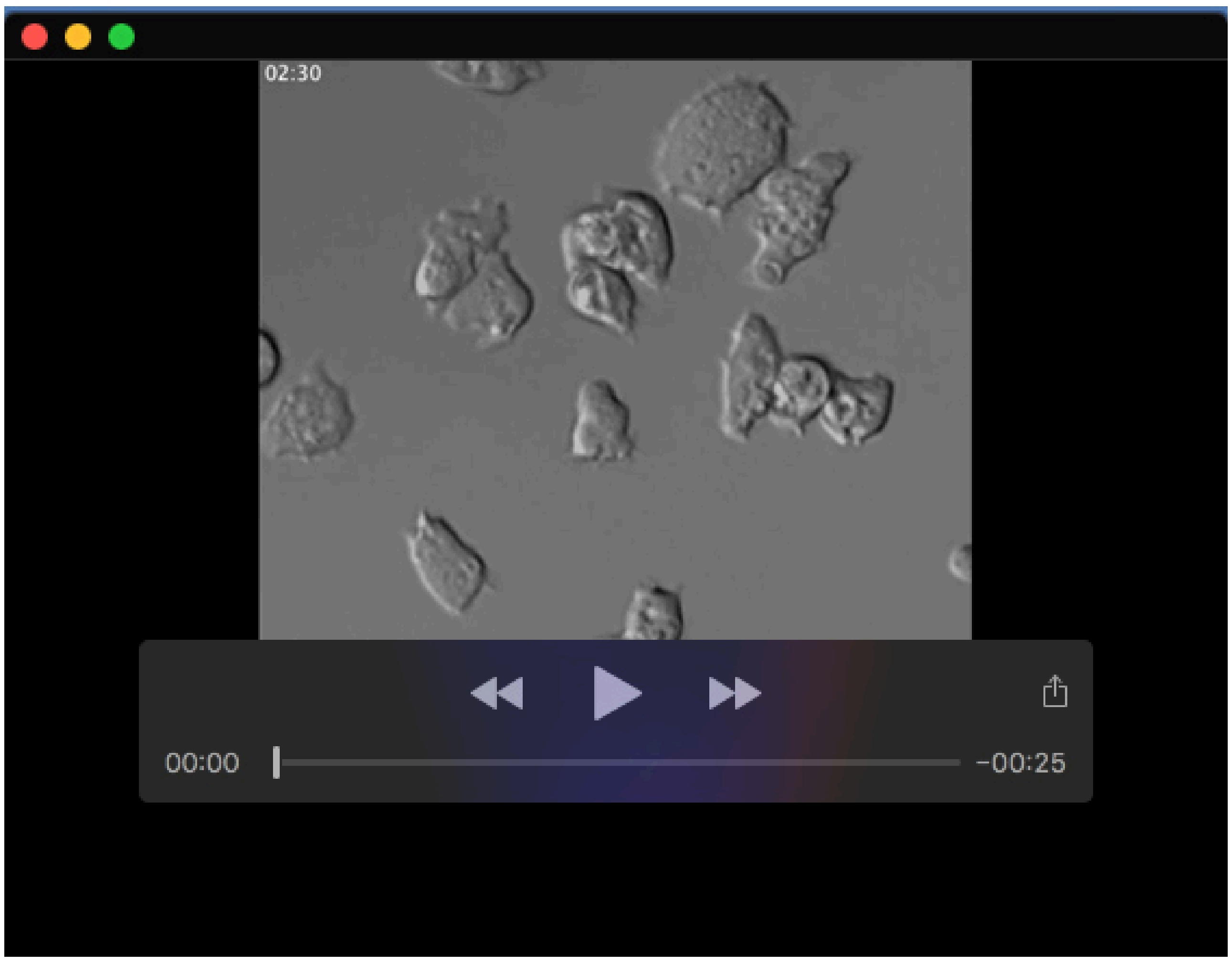


**Movie 2.** rAprA reduces cell motility. Ax2 cells were placed in wells in HL5 and were allowed to adhere for 30 minutes. rAprA was then added to 300 ng/ml, and cells were imaged starting 1 minute after adding rAprA. Elapsed time in minutes:seconds is at upper left. The horizontal field of view is 107 µm. Video is representative of 4 independent experiments.

Journal of Cell Science • Supplementary information

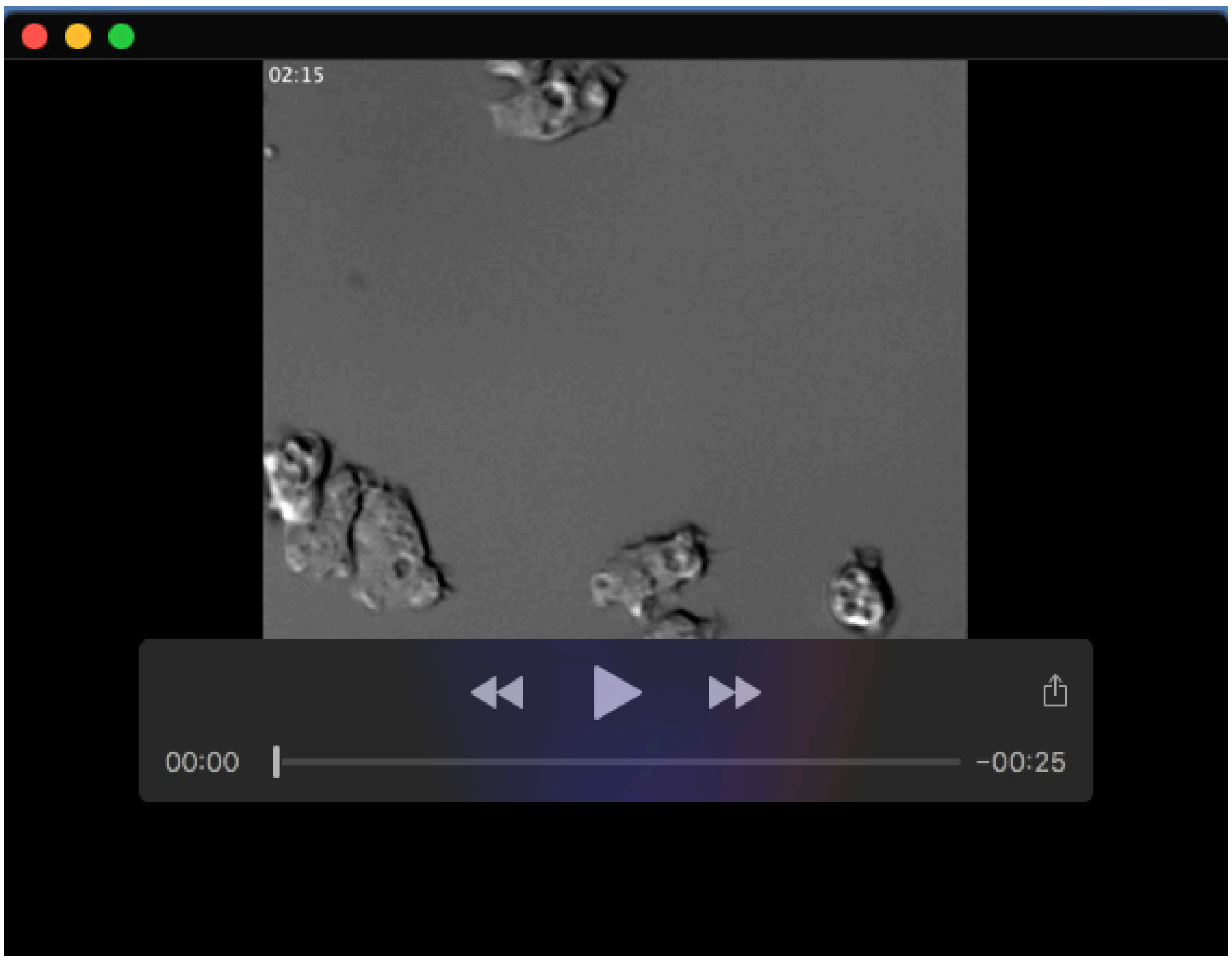


**Movie 3.** Motility of *pipkinA¯* cells in the absence of exogenous AprA. *pipkinA¯* cells were placed in wells in HL5 and were allowed to adhere. After 30 minutes, cells were imaged. Elapsed time in minutes:seconds is at upper left. The horizontal field of view is 107 µm. Video is representative of 4 independent experiments.

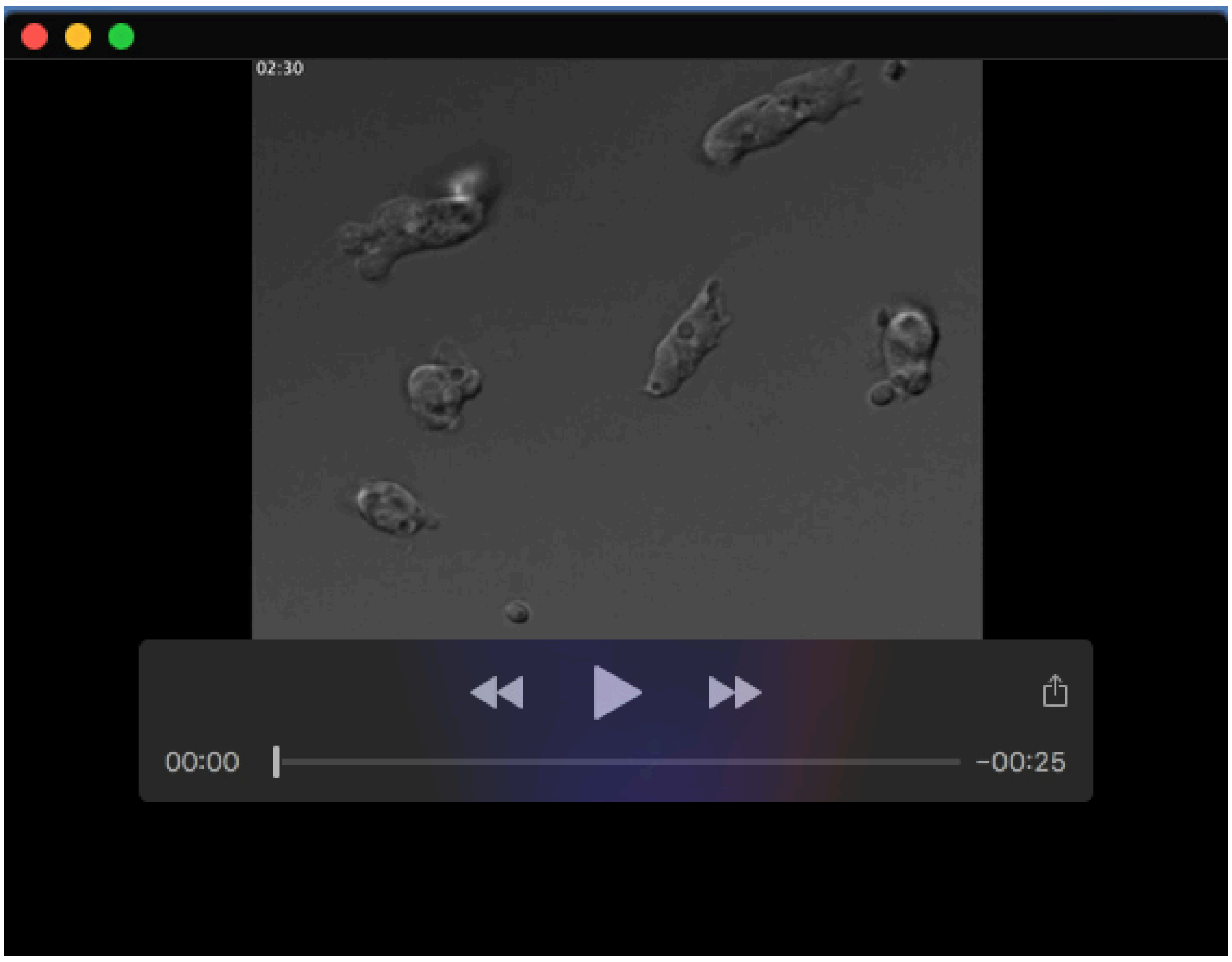


**Movie 4.** rAprA does not reduce *pipkinA¯* cell motility. *pipkinA¯* cells were placed in wells in HL5 and were allowed to adhere for 30 minutes. rAprA was then added to 300 ng/ml, and cells were imaged starting 1 minute after adding rAprA. Elapsed time in minutes:seconds is at upper left. The horizontal field of view is 107 µm. Video is representative of 4 independent experiments.

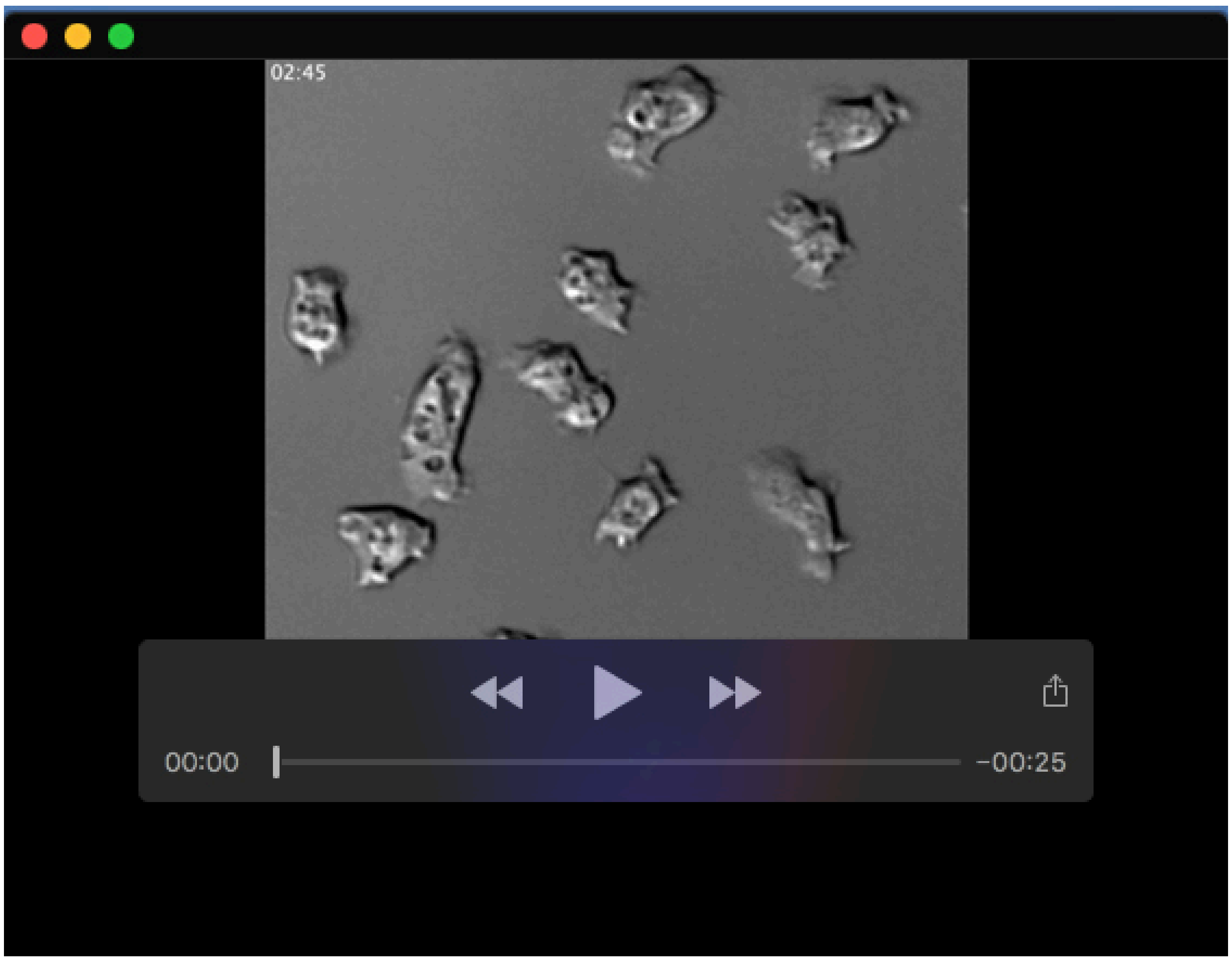


**Movie 5.** Motility of *pipkinA*$^{-}$/ *pipkinA-GFP* cells in the absence of exogenous AprA. *pipkinA*$^{-}$/*pipkinA-GFP* cells were placed in wells in HL5 and were allowed to adhere. After 30 minutes, cells were imaged. Elapsed time in minutes:seconds is at upper left. The horizontal field of view is 107 µm. Video is representative of 4 independent experiments.

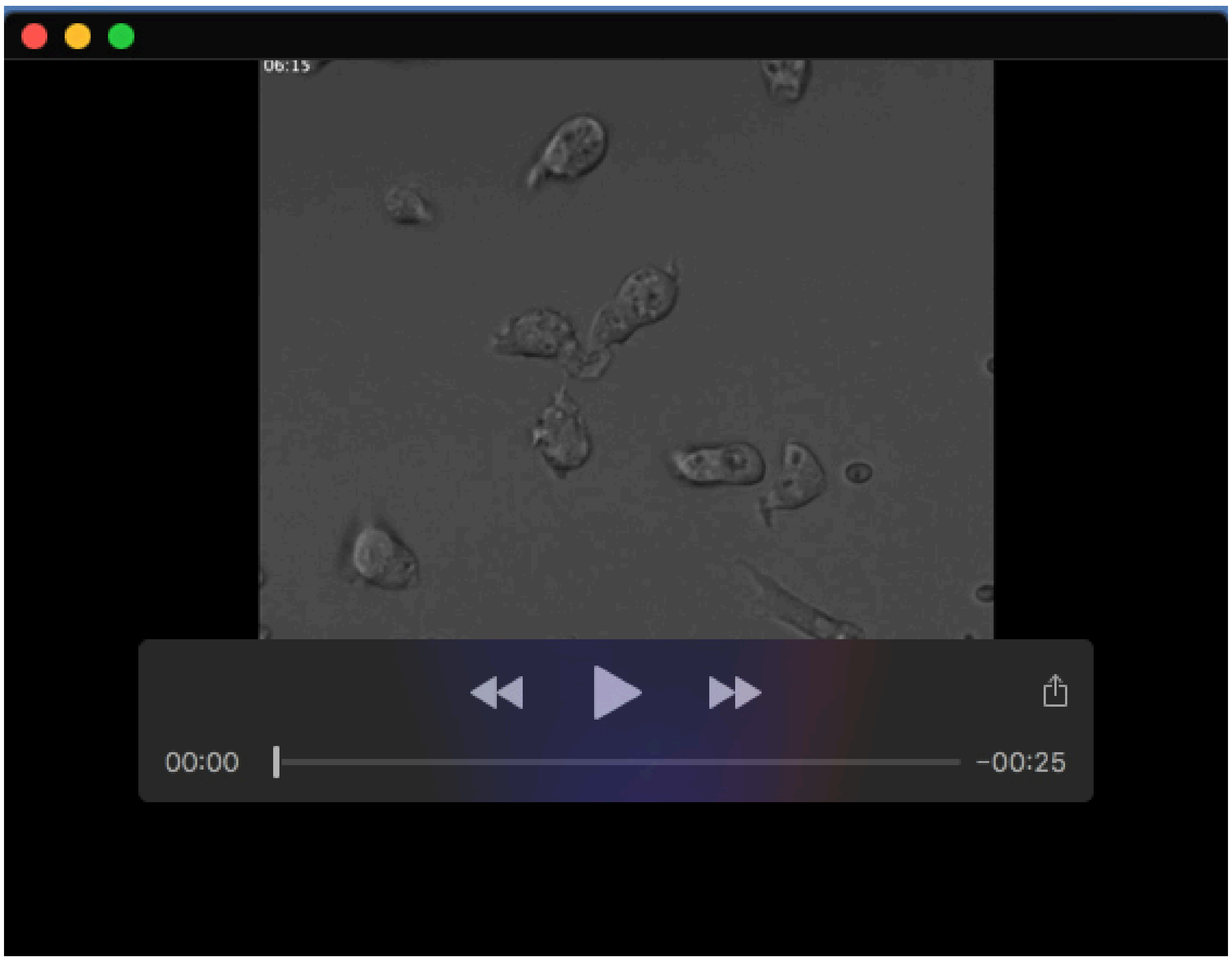


**Movie 6.** rAprA reduces motility of *pipkinA*$^{-}$/ *pipkinA-GFP* cells. *pipkinA*$^{-}$/*pipkinA*$^{OE}$ cells were placed in wells in HL5 and were allowed to adhere for 30 minutes. rAprA was then added to 300 ng/ml, and cells were imaged starting 1 minute after adding rAprA. Elapsed time in minutes:seconds is at upper left. The horizontal field of view is 107 µm. Video is representative of 4 independent experiments.

Journal of Cell Science • Supplementary information
